# Transmission Scenarios of *Listeria monocytogenes* on Small Ruminant On-Farm Dairies

**DOI:** 10.3390/foods12020265

**Published:** 2023-01-06

**Authors:** Dagmar Schoder, Alexandra Pelz, Peter Paulsen

**Affiliations:** 1 Institute of Food Safety, Food Technology and Veterinary Public Health, Unit of Food Microbiology, University of Veterinary Medicine, Veterinaerplatz 1, 1210 Vienna, Austria; 2 Vétérinaires sans Frontières Austria, Veterinaerplatz 1, 1210 Vienna, Austria; 3 Institute of Food Safety, Food Technology and Veterinary Public Health, Unit of Food Hygiene and Technology, University of Veterinary Medicine, Veterinaerplatz 1, 1210 Vienna, Austria

**Keywords:** *Listeria monocytogenes*, direct marketing, farm sales, cheese, dairy products, small ruminant, contamination routes, mastitis

## Abstract

*Listeria monocytogenes* can cause severe foodborne infections in humans and invasive diseases in different animal species, especially in small ruminants. Infection of sheep and goats can occur via contaminated feed or through the teat canal. Both infection pathways result in direct (e.g., raw milk from an infected udder or fresh cheese produced from such milk) or indirect exposure of consumers. The majority of dairy farmers produces a high-risk product, namely fresh cheese made from raw ewe’s and goat’s milk. This, and the fact that *L. monocytogenes* has an extraordinary viability, poses a significant challenge to on-farm dairies. Yet, surprisingly, almost no scientific studies have been conducted dealing with the hygiene and food safety aspects of directly marketed dairy products. *L. monocytogenes* prevalence studies on small ruminant on-farm dairies are especially limited. Therefore, it was our aim to focus on three main transmission scenarios of this important major foodborne pathogen: (i) the impact of caprine and ovine listerial mastitis; (ii) the significance of clinical listeriosis and outbreak scenarios; and (iii) the impact of farm management and feeding practices.

## 1. Introduction

The bacterial genus *Listeria* comprises 21 species of Gram-positive, motile, facultative anaerobic, non-spore-forming rods up to 2 µm in length [1]. Of these, *Listeria monocytogenes* (*L. monocytogenes*) has been studied the most extensively. *L. monocytogenes* is a facultative intracellular bacterium, which can cause severe foodborne infection in humans and invasive diseases in different animal species, especially farm ruminants [2,3]. 

While ruminants, particularly small ruminants, are extraordinarily susceptible to *L. monocytogenes*, other vertebrate wild fauna and birds can excrete the bacteria without notice from their gastrointestinal tracts either continuously or intermittently and for weeks at a time [4]. The widespread occurrence of *L. monocytogenes* in rural environments and its strong association with domestic ruminants make eliminating the risk of listeriosis difficult. Indeed, it is well documented that *L. monocytogenes* is prevalent in the ruminant farm environment [5,6,7,8,9,10,11,12]. However, information on *Listeria* transmission dynamics on small ruminant on-farm dairies is scarce [13,14,15,16]). 

Listeriosis outbreaks in ruminants have been repeatedly reported in the scientific literature and are often referred to as a “silage disease”, as it is strongly associated with the ingestion of spoiled silage [17]. Infected farm ruminants and contaminated agricultural environments rarely appear to directly cause human infection. However, animal-derived food products that are not processed before consumption, such as raw milk, clearly represent a direct link [18]. Minimizing cases of human listeriosis is dependent upon improving our understanding of how to limit contamination of food with *L. monocytogenes*. This is a challenging task, as the pathogen is widely disseminated in nature, has successfully infiltrated both farm and food processing environments, and can enter the food chain at nearly every stage of production [19]. 

The extraordinary viability of *L. monocytogenes* over wide temperature and pH ranges and its ability to survive at high salt concentrations pose significant and ongoing challenges to the food industry and markedly affect the ultimate risk for the consumer [3]. Ready-to-eat foods can be contaminated post-processing (i.e., during portioning, slicing, and packaging). Intermediate moisture and non-acidic foods require refrigerated storage that actually favours the growth of this cold-tolerant pathogen [19,20].

In recent years, significant changes have been noted in the European food market. These essentially reflect increased production of regional and processed farm products. It is clear that, in a competitive marketing environment, farmers are seeking new niche products as prices decrease and various regulations place limits on production [21].

Due to the simplicity of the manufacturing procedure, many ewe and goat dairy farmers produce fresh cheese from raw milk. These milk products have recently become very popular [22]. Growing numbers of people now consume non-pasteurized sheep or goats’ milk or cheese products for practical reasons (e.g., dairy farm families), medical reasons (allergies or intolerance to cow’s milk), or the perceived health benefits of raw milk products. Nonetheless, direct marketers are operators of food businesses and therefore have a responsibility to ensure that the food they market is safe. Furthermore, direct marketers, and everyone involved in the industry, should have a high degree of “quality awareness” and must live by this ideal. 

“Direct Marketing” or “Short Food (Supply) Chain” are terms used synonymously for direct sales from producers to final consumers. Direct marketing in the narrower sense is the sale of agricultural products directly from the farmer to the final consumer. For milk and dairy products, this covers the following distribution channels: (i) farm gate sales; (ii) farm markets or weekly markets; (iii) sales areas outside the farm; (iv) doorstep sales; (v) “new” sales channels (e.g., internet ordering and subsequent shipment of products); and finally (vi) delivery to private households.

Direct marketing in the broader sense of the term includes not only sales to the final consumer but also the following distribution channels: (i) delivery to large customers (canteens, restaurants, etc.); (ii) sales to individual retail outlets, natural food shops, delicatessens, farmers’ shops; and (iii) consumer-producer communities (food co-ops, community-supported agriculture, etc.). However, most farmers operate several forms of direct marketing and there is often no clear distinction between the various forms of marketing [23].

Direct marketing farmers are food business operators and are therefore responsible for the safety of their products from primary production to delivery to the final consumer. Only “safe food” may be placed on the market. In contrast, “unsafe” is defined as harmful to health or unsuitable for consumption [24]. A major contribution to food safety is the implementation of a self-monitoring system. This means that the company must establish a self-control system for its operation [25]. Several manuals have also been developed to implement self-monitoring systems in order to align rules in-force more precisely with the actual requirements for direct marketers [26].

Since 2002, the European Union has issued general (Regulation (EC) No 178/2002, [24]; Regulation (EC) No 852/2004, [27]) and specific regulations concerning the hygiene of foods of animal origin (Regulation (EC) No 853/2004, [28]) and microbiological food safety criteria (Commission Regulation (EC) No 2073/2005, [29]). Subsequently, additional national laws or regulations were set in force for small enterprises with regional activities (e.g., in Austria, [30,31,32]). In principle, the same requirements for food safety apply to any undertaking in which foodstuffs are produced, manufactured, treated and/or placed on the market.

The following hygiene requirements apply to direct marketing farmers: (i) compliance with requirements for buildings and equipment (e.g., sanitary installations, lighting, ventilation equipment, flooring, walls, doors and windows); (ii) the use of appropriate raw material with known origin; (iii) safe handling of food (including packaging and transport); (iv) safe waste disposal; (v) pest control measures; (vi) cleaning and disinfection plans; (vii) water quality; (viii) compliance with the cold chain; (ix) personal hygiene; (x) training and, finally; (xi) application of Hazard Analysis and Critical Control Points (HACCP) principles, including good manufacturing practices and product tests [25,33,34].

Each farmer has his own registered number (for example, the farm and forestry operational information system number (LFBIS number)). Regardless of whether or not checks are carried out on farms under different brand programs, direct marketers—as any other food businesses—are subject to controls by food inspection bodies. Dairy direct marketing farmers are mainly inspected by veterinary control offices. The inspection bodies primarily check whether a suitable self-control system is in place and actually implemented. Food products are examined sensorially and microbiologically, as well as assessed for compliant labelling. 

Generally, small scale on-farm dairies and their self-control systems almost exclusively spotlight their final product and merely identify problems passively as they occur, whereas food business operators, on the industrial scale, must implement a more complex HACCP-based system. Such a system embraces the entire production process proactively by prevention and ensures a consistent quality. In this way, a company can control or mitigate hazards that may arise at any point during the complete production process.

Despite the seemingly endless variety of food items, with more differing foodstuffs than ever being consumed, milk is still an essential basic food for the majority of consumers. However, it also remains a prime nutrient medium for a wide range of pathogens. Information from various large outbreaks importantly demonstrates that milk and milk products have served as the most common vectors for *L. monocytogenes* transmission [35].

Globally milk production is dominated by the dairy cattle sector, which, according to the FAO, accounts for 81% of worldwide production followed by 15% for buffalo and a combined 4% for goats (1.9%), sheep (1.3%), and camels [36]. Although there has been a decline of 8.9% in livestock ruminants across the EU within the last two decades, there was a significant production increase in raw milk on EU farms. 

Another special feature is that in many rural or arid regions, particularly in the Mediterranean area, sheep and goats make an important contribution to the overall milk production. In 2020, according to the “key figures on the European food chain” from the annual report of sectoral and regional statistics, Eurostat, 589,000 to 684,000 tonnes of ewes’ milk were produced in Spain and Greece, whereas the main producer of goats’ milk in the EU was France with 523,000 tonnes milk per year. The report further states that the majority of raw milk production in the EU is delivered to dairies. Still, 10.6 million tonnes were used on farms, being consumed by the farmer’s family, sold directly to consumers, used as feed or processed directly. With 78.7%, Romania holds the highest direct milk-marketing rate, followed by Bulgaria (55.9%). In all other member states, more than 70% of the total milk amount are delivered to dairy companies [37].

It is clear that with 450,000 goat and 850,000 sheep farms the small ruminant sector constitutes just a small share of the total output of the EU livestock sector. However, more than 1.5 million people work on these farms, and sheep and goat rearing takes place mostly on pastureland in remote and disadvantaged rural areas. Thus, the sheep and goat sectors actively contribute to landscape and biodiversity conservation [37]. 

As mentioned above, sheep and/or goat milk and their respective processed products may have many beneficial health impacts, which could appeal to modern consumers [38,39,40]. Sheep and goat milk, due to protein differences with cow milk, induce fewer allergy responses. The levels of minerals, vitamins, and essential fatty acids are also generally higher than in cow milk. Sheep milk has a much higher concentration of conjugated linoleic acid (CLA) than both goat and cow milk [38]. CLA is claimed, for example, to prevent obesity [41] and reduce triglyceride levels. It should, therefore, help to prevent coronary heart disease and atherosclerosis [39]. 

Due to its higher fat and protein content, sheep milk is also particularly beneficial for cheese production [40]. Less additives, such as calcium chloride and rennet, are needed in sheep milk curd production, compared to other ruminants’ milk. 

Taking all of these physical, nutritional, and health benefits into account, it is not surprising that there is a growing demand for milk from small ruminants. In Europe, the production of sheep milk accounted for around 2.8 million tonnes, whereas almost 33% (0.9 million tonnes) were processed directly on-farm into cheese [37]. 

As mentioned above, each direct marketer is a food business operator and, as such, is responsible for the safety of the food that he or she places on the market. Therefore, they must follow good hygiene practices and manage their operations in such a way as to monitor food safety hazards. However, the majority of on-farm dairies produce a high-risk product, which is cheese made of raw milk. The cheese production takes place in direct proximity to animals and the barn environment. Consequently, the microbial contamination pressure on the cheese production environment is classified as “very high”. Yet, surprisingly, almost no scientific studies have been conducted dealing with the hygiene and food safety aspects of directly marketed dairy products. *L. monocytogenes* prevalence studies on small ruminant on-farm dairies are especially scarce. Therefore, it was our aim to focus on this sector and highlight various transmission scenarios of this important major foodborne pathogen. 

## 2. Transmission Scenarios

**Figure 1:**
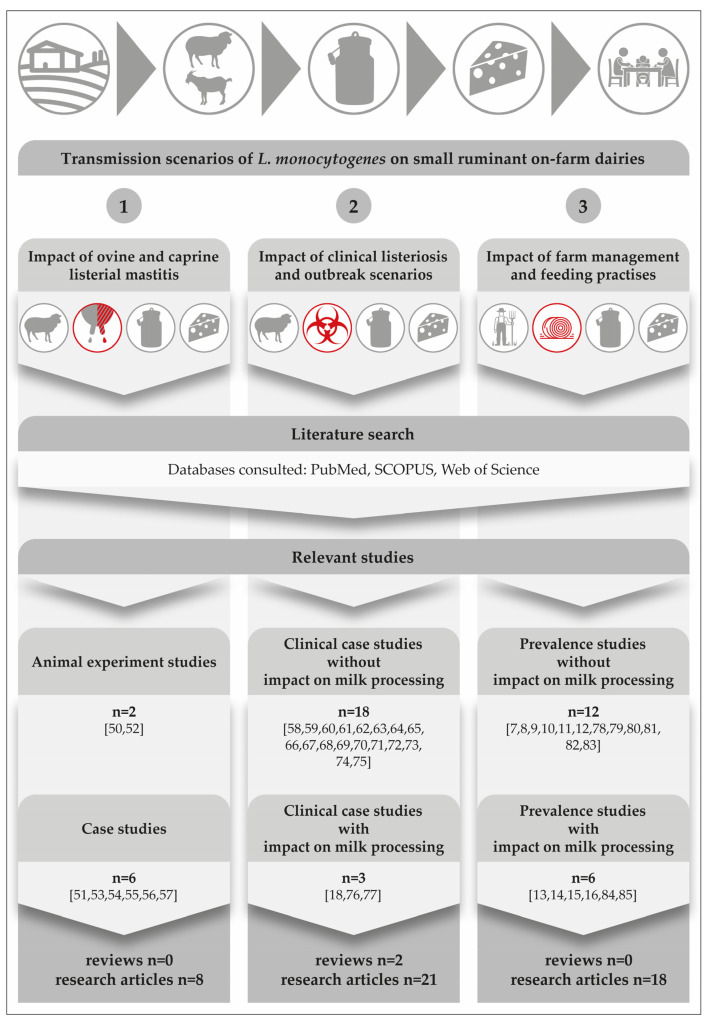
Results from a comprehensive literature search focusing on three main transmission scenarios on small ruminant on-farm dairies. The icons on the top of the figure depict the major steps in the field-to-table continuum [7,8,9,10,11,12,13,14,15,16,18,50,51,52,53,54,55,56,57,58,59,60,61,62,63,64,65,66,67,68,69,70,71,72,73,74,75,76,77,78,79,80,81,82,83,84,85].

In order to elucidate how contamination of dairy products with *L. monocytogenes* occurs on small ruminant on-farm dairies, we performed a systematic literature search in PubMed, SCOPUS and Web of Science databases, for the publication period from 1944 to 2022. The search strategy and the outputs are represented in Figure 1. Basically, we considered the epidemiology of subclinical listerial mastitis (scenario 1) and clinical listeriosis (scenario 2) and the impact of farming-, feeding- and milk processing practices (scenario 3). 

### 2.1. Transmission Scenario 1: Impact of Ovine and Caprine Listerial Mastitis 

*L. monocytogenes* can colonize the mammary complex of ruminants. Although *L. monocytogenes* is common in the faeces of ruminants and widespread in the environment [4], only a few cases of bovine [42,43,44,45,46,47,48,49] and ovine [50,51,52,53,54,55,56] listerial mastitis have been reported. We could retrieve merely a single study on caprine mastitis [57]. Interestingly, listerial mastitis has not yet been covered in any review article (Figure 1). 

Another surprising detail is that there is not a single study from America, Asia, Australia, or Africa; all listerial mastitis studies were performed in Europe. This fact is quite remarkable, if one considers that there was a dramatic increase especially in dairy goat production during the past decade, with Asia seeing the largest growth of 22%, followed by Africa (13%), Oceania (9%), and America (5%) [86].

A comprehensive search of the literature revealed two experimental studies on the course of listerial mastitis in small ruminants (Figure 1). They have shown that inoculations of 300 CFU to 1000 CFU of *L. monocytogenes* into the udder of ewes are sufficient to result in mastitis [45,52]. All inoculated ewes became infected and developed chronic subclinical mastitis, regardless of the serotype or origin of the strains used. According to Tzora et al. [52], only one single ewe out of 34 animals showed typical signs of acute clinical mastitis immediately after the inoculation. The gland was larger and hotter and its secretion contained clots. There was also an increase in the internal body temperature.

The somatic cell count of all infected sheep was always greater than 1.0 × 10^6^ cells per ml and *L. monocytogenes* could be consistently isolated from the milk over a period of 88 days. *L. monocytogenes* was also detected from the mammary lymph nodes, but not from any internal organ of any inoculated ewe. Histologically, in the early stage of the infection, extra-alveolar neutrophilic infiltration and interstitial oedema were predominant. Subsequently, 25 days after inoculation, chronic inflammatory signs predominated, such as destruction of alveoli and fibrous tissue proliferation, with lymphocytes as the main cell type [52]. The findings of both studies provide clear evidence that *L. monocytogenes* is pathogenic for the ovine and caprine mammary gland.

With regard to naturally occurring cases of ovine mastitis (Figure 1), it is worthwhile to compare a Greek study from Fthenakis et al. [51] with the findings of an Austrian research team [53,54,55]. Fthenakis et al. [51] monitored the udder health, somatic cell count and the shedding of *L. monocytogenes* in 98 ewes. Half udder milk samples were collected at three separate time points during the lactation period, including: (i) 15 ± 30 days post- lambing; (ii) 6 ± 7 weeks after initial sampling; and (iii) 6 ± 7 weeks on from collection of the second sample. There were diagnoses of clinical mastitis in any of the ewes, though the prevalence of subclinical *L. monocytogenes* mammary infections was 3.1% during collection of the first and the second samples and this had increased to 6.2% by the third time point. Examination of the milk of ewes with mammary infection revealed somatic cell counts ranging from 1.8 × 10^6^ to 3.0 × 10^6^ cells/mL. Furthermore, *L. monocytogenes* could also be detected in the faeces of 19.4% of the animals. The authors concluded that infection of the mammary gland with *L. monocytogenes* had occurred via the bloodstream. Firstly, there was an 83% higher prevalence of bilateral mammary infection and, secondly, the pathogen was isolated from the liver of two of the four infected ewes. These findings are only partly in accordance with other case studies, which consider intramammary infection to be the most likely and emphasize that *L. monocytogenes* has to contaminate the teat before penetration into the udder [45,53,54,55,56,57].

Briefly, Schoder et al. [53,55] studied two cases of ovine *L. monocytogenes* mastitis over a period of 7 months. On a daily basis, the animals were clinically examined. After adspection and palpation of the mammary gland, the California mastitis test (CMT) was performed and half udder milk samples were collected. During the entire observation period, the animals continued to eat well and did not show any signs of distress or evidence of systemic reaction. The milk appeared to be normal, was not discoloured and did not contain any flakes or clots. However, CMT showed consistently thick gel (++) or thick and sticky gel (+++) reactions. Somatic cell counts averaged ≥ 10^6^ per ml milk. Both sheep shed *L. monocytogenes* at a mean concentration of 3.8 × 10^4^ (range 9.0 × 10^1^ to 4.0 × 10^5^) and 2.2 × 10^4^ (range 1.3 × 10^3^ to 8.1 × 10^4^) CFU/mL, respectively. Subclinical mastitis was diagnosed without palpable changes in the consistency of the udder parenchyma. 

The histopathological and immunohistochemical findings revealed that chronic inflammatory features predominated [54,57]. There was a diffuse infiltration with lymphocytes, plasma cells and macrophages. Additionally, alveolar destruction and proliferation of fibrous tissue were recorded with a very strong immunoreactivity for CD5 cells. 

*Listeria* could be cultivated from the mammary parenchyma of the infected halves and from the lymph nodes [52,54,57]. In contrast to the Greek study, all other internal organs showed no abnormalities, and no single *Listeria* could be isolated [54,57]).

**Figure 2:**
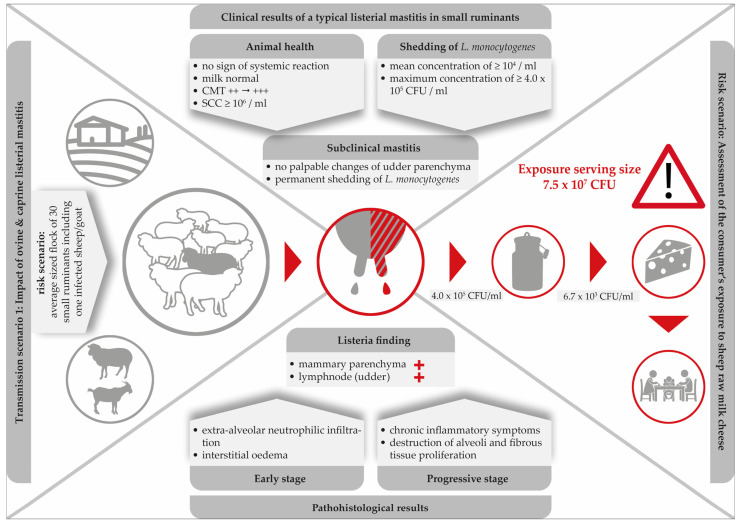
Clinical and histopathological findings in a typical listerial mastitis in small ruminants and consequences for the safety of cheese produced on-farm.

Literature findings suggest that caprine and ovine mastitis are very much comparable. Furthermore, the typical listerial mastitis in small ruminants is defined: (i) by its subclinical nature; (ii) a high somatic cell count (≥10^6^ SCC per ml); (iii) persistent shedding of the pathogen bacteria; (iv) by induration and atrophy of the mammary parenchyma in progredient stages of the infection and, finally; (v) the local invasion via the teat canal seems to be the most likely route of infection. Figure 2 illustrates the main clinical and pathological findings of listerial mastitis in small ruminants. A risk scenario was designed to highlight the dimension of the consumers’ exposure.

Clearly, mastitis attributed to *L. monocytogenes* is especially dangerous due to its subclinical nature. While milk from infected udders remains visually unchanged and the udders show no clinical signs, *L. monocytogenes* continues to be shed up to concentrations of 4.0 × 10^5^ CFU/mL [53]. With respect to food safety, listerial mastitis has two main consequences: firstly, the direct contamination of bulk milk and raw milk cheese with high loads of the pathogen and, secondly, the increase of environmental colonization of the farm and the cheese processing environment. Furthermore, within the last decades, hypervirulent *L. monocytogenes* strains were found to be significantly associated with subclinical mastitis and were more commonly isolated from dairy products [87]. 

Remarkably, merely three single studies have been published demonstrating the consequences of ovine listerial mastitis on the further processing of milk to cheese [53,55,56]. Based on two cases of ovine mastitis, a risk scenario was designed in order to assess the consumer’s exposure to *L. monocytogenes* per serving size of sheep raw milk cheese [55]. Various cheese-making procedures were performed. The results were alarming: the final level of contamination was up to 7.5 × 10^7^ CFU/serving size. Certainly, such an extremely high dose qualifies the cheese to be hazardous for consumers (Figure 2). 

Clearly, there is an urgent need to screen small ruminant farms for the presence of cases of subclinical *L. monocytogenes* mastitis by implementing CMT at least once per week [53]. With regard to caprine mastitis, however, milk SCC is a less reliable indicator of inflammation than in other dairy animals [88]. Therefore, the routine control of subclinical mastitis cases by SCC monitoring, such as with the CMT, is less meaningful than in cows or ewes [57]. In conclusion, Addis et al. [57] emphasized that the milk of all goats of a dairy farm should be screened for the presence of *L. monocytogenes* on a regular basis. 

Together, these data suggest that small ruminant dairy farms, which sell milk and/or cheese made of raw milk directly to consumers or retailers, are in urgent need of an efficient monitoring program for the detection of *L. monocytogenes*. 

### 2.2. Transmission Scenario 2: Impact of Clinical Listeriosis

*L. monocytogenes* is a globally distributed pathogen with the ability to cause disease in a wide range of animal species, though sheep are particularly susceptible to infection. In the northern hemisphere, infections are typically seasonal and most common sporadically in winter and early spring in association with silage feeding. Meanwhile, in the southern hemisphere, most listeriosis cases in ruminants occur during the warmest months of the year and the transition from rainy to dry season. It can be assumed, that not only silage, but also feedstuff and water generally play an additional role in the mode of infection [17]. 

**Figure 3:**
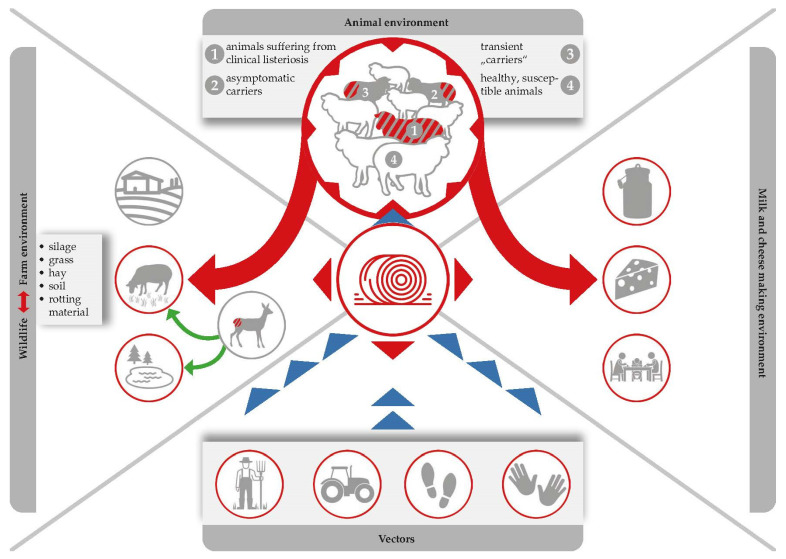
Silage serves as the most important *Listeria* reservoir. Ingestion of contaminated feed leads to the pathogen multiplying within animal hosts, and the bacteria are then excreted via faeces, which are in turn used as fertilizers, which forms a recurring cycle that favours the persistence of *L. monocytogenes* in both farm and natural environments.

Listeriosis of small ruminants is well documented and there is a numerous number of case studies, including two comprehensive review articles. However, case studies focusing on the impact on milk processing are scarce [18,76,77] (Figure 1). The disease is clearly and most commonly caused by oral infection, but other entrance sites, such as the conjunctiva, microlesions of the skin, buccal and genital mucosa, or the teat canal have also been described [89]. After oral infection, *L. monocytogenes* is able to colonize the gastrointestinal tract. Animals either become asymptomatic carriers or they develop mild symptoms of a self-limiting enteritis. In both cases, the bacterium is shed with the faeces and is able to heavily contaminate the farm and milk processing environment (Figure 3), [4]. The interplay of environmental reservoirs outside the farm and of vectors and the farm animals is shown in Figure 3. Notably, the excretion of *L. monocytogenes* by the farm animals is not only a food safety and herd health issue, but can also contribute to infection of wildlife. 

Interestingly, there is a study describing the case of an orally infected sheep that carried *L. monocytogenes* in the spleen, liver and lymphoid organs without showing any clinical symptoms. The authors concluded that *L. monocytogenes* intestinal infection and translocation to visceral organs may occur asymptomatically [90]. Additionally, in the case of invasive listeriosis, the pathogen is able to cross the gastrointestinal barrier causing severe illnesses including abortion, septicaemia and rhombencephalitis—the so-called “circling disease”—which accounts for the vast majority of invasive clinical infections in small ruminants [17,89].

The incubation period in small ruminants varies according to pathogenesis. It can be as short as one to two days for septicaemia or gastrointestinal forms, two weeks for abortion, and between four and six weeks for the encephalitic form. The pathogen has a particular affinity for the central nervous system in sheep. The main clinical signs include apathy, fever, anorexia, head pressing or compulsive circling and unilateral or bilateral cranial nerve deficits. *L. monocytogenes* ascends the nervous system following peripheral traumatic lesions (e.g., ascending intra-axonal migration within the trigeminal nerve or other cranial nerves following small lesions of the buccal mucosa). Another route involves ascending infection via the sensory nerves of the skin [17].

Neurological symptoms leave little doubt as to their cause and affected animals can be removed from herds. The milk and meat of these affected animals is rather unlikely to enter the food chain [18,76,77]. However, especially during an outbreak event, massive contamination of the animal environment, both through contaminated feed and faecal shedding from exposed animals, may lead to cross-contamination of: (i) the milk processing and cheese-making environment; (ii) bulk tank milk; and (iii) subsequently, the cheese products themselves (Figure 3).

Following an outbreak of clinical listeriosis in sheep, Wagner et al. [77] sourced the infectious agent to grass silage feed, which was contaminated with 10^5^ CFU/g *L. monocytogenes*. The investigation took place on a dairy farm producing raw milk cheese made of 50% ewe and 50% cow milk. Dairy cows were not affected by this outbreak, reflecting the high susceptibility of sheep to listerial infection. Interestingly, the clinical manifestation within the flock of 55 sheep was also quite variable. Although they were all fed from the same batch of silage, only one ewe was affected by central nervous symptoms caused by rhombencephalitis, four ewes suffered from septicaemia and a further nine animals delivered a combined total of 20 stillborn mature foetuses. 

From the animal that had developed central nervous symptoms, *L. monocytogenes* could neither be recovered from the visceral organs nor in the faeces, but was found in a blood sample taken directly from the heart, brain and the nasal mucosa. The authors concluded that the infection had originated within the nasal mucosa and spread to the brain, but that the liver, spleen and other visceral organs remained clear. The route of infection in the other animals was most likely via feedborne transmission. Those with septicaemia suffered from accumulation of *Listeria* in the liver, spleen, heart and lung, with a median concentration of 5.9 × 10^5^ to 6.4 × 10^6^ CFU/g. *L. monocytogenes* could also be detected in the foetal liver, spleen, lung, heart and brain with values ranging from 3.1 × 10^3^ to 5.6 × 10^5^ CFU/g.

Samples from both the farm environment and the cheese production chain, which were randomly taken from ewes, cattle and all individuals who lived on the farm, were positive for *L. monocytogenes,* including 62% of faecal samples and the bulk tank of the cows. Interestingly, one farm worker tested positive for an isolate that was so similar to the outbreak clone that it could not be distinguished genetically, which clearly occurred through them consuming contaminated raw bovine milk. Due to intensive consultation and the fact that the most important countermeasures were immediately taken (silage had been discarded, affected animals had been separated and cleaning and disinfection of the cheese making facilities were implemented), *L. monocytogenes* was not detected in the cheese samples [77]. 

### 2.3. Molecular Epidemiological Aspects of Listeria monocytogenes

Bagatella et al. [17] provides a comprehensive overview of epidemiological and experimental studies, which highlight the genetic heterogeneity of *L. monocytogenes* in humans and in ruminants. A heterogeneity, which is likely linked to the variability observed in virulence and in clinical manifestations, as well as to the environmental distribution of listeriosis [91,92,93]. Research is currently ongoing in an attempt to identify the bacterial determinants driving variability and niche adaptation in *L. monocytogenes*, as well as the principally associated mechanisms [94]. Several bacterial subtypes have been characterized and efforts made to associate them with particular niches and relative virulence. Of the 13 serovars identified, types 1/2a, 1/2b, and 4b were those most frequently found in clinical isolates from both humans and animals. Meanwhile, in cases of ruminant neurolisteriosis and in major outbreaks of listeriosis, serotype 4b was the most dominant [59,66,95,96,97].

All 3 serotypes, apart from being implicated in disease, were additionally isolated from food, food processing and farm environments, and animal faeces [46,98,99,100]. Isolates can be linked to clinical outcomes, the environment and foods through molecular typing methods, including pulsed field gel electrophoresis, multilocus sequence typing (MLST) and whole genome sequencing (WGS). Using these techniques, four distinct lineages (I–IV) were identified and further subdivided into clonal complexes (CCs) and sequence types (STs), or sublineages (SLs) and core genome MLST types (CTs), respectively [101]. 

*L. monocytogenes* that can be frequently isolated from diverse sources are binned into two major lineages (I, II), with lineage I being overrepresented in human clinical isolates and ruminant neurolisteriosis cases as well as being the most genetically homogeneous, while *L. monocytogenes* that are sporadically isolated from animal infections are binned into two minor lineages (III, IV) [63,93,97,102,103,104].

Several CCs were found to be hypervirulent in experimental models, including CCs from lineage I belonging to serotype 4b (such as CC1, CC2, CC4, and CC6), these were also significantly linked to human clinical cases and well-adapted to host colonization compared to clones overrepresented in food and the environment (such as CC9 and CC121) [91,92,103].

Within clinical isolates and particularly neurolisteriosis isolates from ruminants, lineage I, specifically CC1 and CC4, were found to be significantly overrepresented compared with other clinical listeriosis syndromes in ruminants, such as abortion, mastitis or gastroenteritis. Additional isolates, from diseased animals and diseased animal environments, that are commonly found include isolates from both lineage I (CC2, CC217, CC6, CC191, CC59) and lineage II (CC7, CC11, CC14, CC37, CC204, CC412) [63,87,93,100].

We can conclude that preventing disease in ruminants and its concomitant transmission to humans is a challenging task, requiring efficient surveillance and control measures. As ruminants, humans and the environment are indelibly connected, achieving a more comprehensive understanding of the pathogenesis of listeriosis and its molecular epidemiology within these domains is critical for developing methodologies to meet the challenge in congruence with the “Farm to Fork” strategy and One Health concepts.

### 2.4. Transmission Scenario 3: Impact of Farm Management and Feeding Practices

Transmission of foodborne pathogens frequently involves complex interactions among the pathogen, the environment and one or multiple host species [105]. *L. monocytogenes* is a ubiquitous pathogen that can be found in moist environments, soil, water and decaying vegetation [106]. However, does *L. monocytogenes* still have its origin and main habitat in the natural environment and wildlife? Or, can we assume that this major pathogen acts as a cultural successor, which has already successfully colonized the farm- and food-production environment, creating new reservoirs there? Interestingly, the prevalence of *L. monocytogenes* in the dairy cattle environment is well documented [5,6]). There are also numerous studies focusing on the occurrence of *L. monocytogenes* in small ruminant farms. However, the knowledge of *Listeria* transmission dynamics and ecology in on-farm dairies is limited (Figure 1). 

As far as we know from literature, *L. monocytogenes* prevalence is normally lower during the pasture season than it is during the indoor season [107,108]. Furthermore, the pathogen has been isolated from both clinically infected and clinically symptomless ruminants. In fact, *L. monocytogenes* can be shed by (i) healthy sheep and goats (so called transient “carriers” and asymptomatic carriers); and (ii) by ruminants suffering from a clinical listeriosis (Figure 3). 

Faecal shedding of *L. monocytogenes* has several effects on food safety: (i) *L. monocytogenes* accumulation within the immediate environment of the barn increases the probability that more animals will become infected; (ii) contamination of feed and crops with *L. monocytogenes* can occur when the manure of infected animals is used as fertilizer in agriculture, whilst water sources can be contaminated by runoff from farms [109,110]; (iii) raw milk contamination may occur due to poor hygiene standards during the milking of animals in which infection has gone undetected (Figure 3).

Ingestion of contaminated feed, multiplication of the pathogen in animal hosts, and subsequent excretion of the bacterium via faeces, which are in turn used as fertilizers, form a recurring cycle which favours the persistence of *L. monocytogenes* (Figure 3), [5]. It cannot be denied that there is a high contamination pressure of *L. monocytogenes* on dairy farms and we have to admit that the problem is entirely self-generated. Alarmingly, *L. monocytogenes* may be present in 8% up to as much as 50% of faecal samples collected from dairy sheep and goats [10,13,18,78]. The shedding itself is associated with animal stress and is strongly connected to the contamination of silage [111]. While *L. monocytogenes* is rarely detected on growing grasses prior to processing, detection rates in clamp silages range from between 2.5% and 5.9% and reach up to 22.2% in large bales. This further increases to an alarming 44% in mouldy silage samples [112]. Alternatively, use of inadequately fermented silage (pH of 5.0 to 5.5) contaminated by soil and tainted crops can permit subsequent amplification of *L. monocytogenes* numbers to high levels. In this way, field studies consistently highlight silage feeding as the main factor associated with farm animal exposure. However, the pathogen could also be isolated from a number of other sources, including bedding material, feed bunks, and water troughs [113,114]. 

Once ingested via feed, *L. monocytogenes* transforms its metabolism and colonises the ruminant gastrointestinal tract intracellularly as a cytosol-adapted pathogen, thereby escaping immune defence. According to Zundel and Bernard [90], *L. monocytogenes* multiplied in the rumen of sheep who were asymptomatic carriers due to the favourable environment of the organ (pH 6.5–7.2 and body temperatures from 38.0 to 40.5 °C). Thus, the rumen content serves as an important reservoir for *Listeria*. 

However, there is still the widespread opinion that grass and soil are initially contaminated by wildlife such as deer and birds, which means that dairy farm animals are mainly subsequently challenged with *L. monocytogenes*, either during grazing or after consumption of silage: Indeed, asymptomatic carriage of *L. monocytogenes* is thought to be prevalent in up to 36% of wild birds. This includes a variety of species, such as crows, gulls, pheasants, pigeons, rooks, and sparrows [4]. Interestingly, it was suggested that birds may be somewhat responsible for spreading strains of *L. monocytogenes* within the human food chain, as there were often similarities in the pulsotypes isolated from the birds with those found in the food chain [115]. However, the findings do not explain if the birds are infected when feeding on fertilized fields contaminated with *Listeria*, if birds contaminate the environment or if both situations apply. Additionally, a wide range of mammals, such as red fox (3.5%), wild boars (25%), and deer (42%), also harbour *L. monocytogenes* [4]. Silage winter feeding is a common practice for free-living [116,117]) as well as farmed [118] wild ruminants in alpine regions, and it remains to be explored to what extent this practice contributes to *Listeria* infections in wildlife. Again, there is considerable evidence that the high prevalence rate in wild animals is entirely self-generated.

Finally, faecal transmission of *L. monocytogenes* is not exclusively driven by animals, either wild or domestic, as it has been shown to occur regularly in humans also [4]. A number of studies have investigated such transmission within specific occupational groups. Laboratory technicians had a 77% high cumulative prevalence rate of faecal carriage. However, prevalence was also very high (62%) in office workers, who were not occupationally exposed to *L. monocytogenes* [119]. Furthermore, 16% of swab samples from the hands of farmers [120] and 5.7% of swab samples from hands and working clothes of abattoir workers [121] were positive for *L. monocytogenes* [4].

Faecal shedding of *L. monocytogenes* by asymptomatic farm animals increases its presence within the farm environment, which leads to an increased risk of feed and food contamination (Figure 3). Therefore, the ecology of *L. monocytogenes* within the agricultural environment should be thorough analysed and *Listeria* reservoirs should be identified and removed as part pathogen reduction programs [99].

Hence, in order to gain a more comprehensive understanding of the transmission dynamics and ecology of *L. monocytogenes*, a prevalence study was conducted in the dairy-intensive region of Austria, focusing on small ruminant on-farm dairies [13]. The study focused on dairy farms that manufactured cheese from raw caprine and ovine milk, and aimed to identify the routes of transmission of *Listeria* spp. and to investigate the link between *L. monocytogenes* mastitis and the contamination of raw milk. A total of 5799 samples were taken from 53 Austrian dairy farms, and the pathogen was found in 0.9% of them. However, none of the samples taken from the udders of the sheep or goats tested positive, meaning that raw milk contamination was not significantly impacted by listerial mastitis.

The prevalence levels from swab samples of working boots and faecal samples were 15.7% and 13.0%, respectively. The investigators concluded that silage feeding practices correlated significantly with the prevalence of *L. monocytogenes* in the farm and milk processing environments. Again, silage was a main culprit, such that *L. monocytogenes* was between three to seven times more likely to be present in farms that fed silage to animals year-round than in farms that did not use silage [13].

Appraisal of state-of-the-art studies now leads us to conclude that silage and the rumen itself serve as the most important *Listeria* reservoirs. While the pathogen persists in a cyclic infection (from faecal excretion to contamination of feed to multiplication in the gastrointestinal tract) [5], it can enter the food chain either by contaminating raw milk or by being excreted from the udder of an infected animal. In turn, this contamination can spread silently to the milk and cheese processing environment (Figure 3); once contaminated, milk and cheese processing devices and premises can act as a reservoir for *Listeria* and contaminate product batches that were originally free from the pathogen.

## 3. Risk Factor: Consumer Habits

### 3.1. Trends in Food Supply and Consumers’ Preferences

As the availability and variety of foods in developed countries have increased over the past several decades, consumer perceptions of these essentials are also changing. Perhaps the greatest influence on European eating habits in modern times was the widespread introduction of efficient and affordable domestic refrigeration in the 1960s. For instance, a majority (58%) of British households owned an electric refrigerator by 1970 [122]. Together with social changes at that time, in the context of post-war reconstruction in Europe, where more women entered the workforce, this led to the growth of supermarkets. Shopping for food each day was no longer necessary. Wartime and rationing survivors, as well as baby boomers, began to enjoy ongoing food abundance.

In the meantime, consumers throughout industrialized countries are becoming increasingly alert to the environmental, social and health consequences of mass-produced, refined foods and the globalization of food production and trade. Opinions now abound as to how further environmental damage by mass agriculture can be prevented, how food production can become sustainable without long transportation distances and how to maintain local economies [123].

These everyday messages are motivating a significant number of people to prefer foods that have been produced in a transparent and sustainable way, that are free from pesticides, agrochemicals, processing contaminants, produced without genetic manipulation, and ideally sourced locally. Perceptions that such food tastes better than superstore alternatives and comes without plastic packaging are self-motivating. These sentiments reasonably converge on the local farms, farmers’ markets or street stallholders as opposed to the local supermarket.

The trend to shop for locally harvested food is likely to increase due to our growing love of organic produce, renewed enthusiasm for vegetarianism and veganism and simultaneously calls for less meat consumption to slow climate change and improve health. Such calls have been advocated by, among others, the Intergovernmental Panel on Climate Change [124] and the World Scientists’ Warning to Humanity [125]. An increasing number of people are vegetarian, vegan, or flexitarian—those who adopt a predominantly plant-based diet with occasional meat consumption.

All of this is intensified by the actions of farmers to keep their businesses viable. It is noteworthy that the number of farms in the EU is in steep decline [37]. There can also be subsidies for farmers to diversify their activities from national and international bodies in the context of development programs for weakened rural communities. Local food systems are purported to promote sustainability, improve local economies, increase access to healthy foods, improve local diets [126] and encourage entrepreneurship and innovation. Direct sales from farm producers to consumers, which include farm gate sales, farmers’ markets and internet-direct marketing, are becoming new marketing channels that retain profits in local communities [127]. Indeed, some forms of direct marketing are integrally linked with tourism in local communities. The complement is that farmers who sell products directly to consumers can attract a number of visitors to a community [128].

To demonstrate an alternative market, farmers’ markets generally also create a context for closer social ties between farmers and consumers—a human connection—but they remain fundamentally rooted in community relations [129]. The obverse, according to Hinrichs, is a distant and anonymous relation between consumers and a few seemingly unpeopled yet powerful transnational corporations. As for the farmers, the higher costs associated with direct marketing can be compensated for by higher revenues from higher prices and reduced uncertainty, which encourages them to enter into quality food projects without investing excessive labour or capital [130]. Farmers can attract premium prices with minimum costs for handling, transportation, refrigeration, storage and retail premise overheads.

These consumer-led changes are certainly encouraging for consumer wellbeing and the planet. However, the production of industrially produced foods can be regulated legally in the interests of consumer health, while this is not so easy to ensure on the smaller scale.

### 3.2. On-Farm Dairies and Raw Milk Consumption

The concept of “produce, sell and buy local” has also resulted in an increased interest in the consumption of raw milk [131]. Raw milk advocates argue that it is a complete, natural food containing more amino acids, antimicrobials, vitamins, minerals and fatty acids than pasteurized milk. Furthermore, raw sheep and goat milk is seen to be a better choice for those with lactose intolerance, asthma, and autoimmune and allergic conditions [38,39,40]. It is estimated that 35–60% of farm families and farm employees consume raw milk on a regular basis, whereas the consumption of raw milk by the urban community is more difficult to estimate [132]. However, raw milk and milk product consumption pose a significant health risk associated with ingestion of *L. monocytogenes*. Surveys from various countries monitoring the presence of this major foodborne pathogen in raw bovine milk (including in-line milk filters), have shown prevalence levels as high as 13% [132]. Studies referring to small ruminant milk revealed a prevalence of up to 17% [133], with the prevalence of pathogens in milk being influenced by numerous factors, including farm size, number of animals on the farm, hygiene, farm management practices, milking facilities, and season [13].

Not surprisingly, numerous foodborne outbreaks caused by milk products contaminated with *L. monocytogenes* are reported [35]. Interestingly, to our knowledge there is no documented case of an outbreak scenario due to cheese manufactured at on-farm dairies. However, Bellemare et al. [134] claimed that the emergence of farmers’ markets in the USA increased the number of outbreaks and cases of foodborne illnesses. They detailed a positive relationship between the number of farmers’ markets per million individuals and the number per million of reported total outbreaks and cases of foodborne illness in the average state by year.

### 3.3. Management of Foodborne Hazards in On-Farm Dairies

For its part, the EU has attempted to reduce food safety risks through programs such as “Farm to Fork” food safety legislation [135]. A broad weakness, however, is that farmers’ markets, for example, tend to be less rigidly regulated than bricks and mortar shops. Consequentially, this opens up the potential for new routes of food contamination that have until now been neglected. Notwithstanding, several EU countries have developed legal frameworks and incentives to support these so-called “short food (supply) chains” [136]. As direct marketing of food from producers to consumers in Europe grows in popularity, we also must be vigilant about new patterns and scales of food contamination.

One of the largest developments in recent years in nutrition is that consumers are increasingly demanding minimally processed, ready-to-eat (RTE) foods that can be stored in refrigerators for up to several weeks. These foods are challenging hygienists’ attempts to ensure microbiological quality and safety [20], not least due to the fact that domestic refrigerators are usually not cold enough [137].

*L. monocytogenes* is psychrotrophic, which means it is able to multiply even at a few degrees above zero. Nevertheless, in general, optimal storage temperatures of 4 °C will usually slow growth of *L. monocytogenes* and may restrict amounts in food to non-harmful doses. However, in the context of a multinational outbreak, the psychrotrophic growth potential of *L. monocytogenes* can be dramatic [138]. The contamination levels of *L. monocytogenes* in lots of acid curd cheese that caused a listeriosis outbreak, which led to a total of 8 deaths among 34 clinical cases, were determined. Contamination levels varied from ≤ 10^2^ CFU/g to 8.1 × 10^8^ CFU/g. Interestingly, contamination levels of ≤10^2^ CFU/g were even found in three of the sixteen lots that had been stored under optimal conditions since the beginning of their shelf-life. Nevertheless, by the end of the shelf life, the contamination levels were found to have increased to the health-endangering levels of 10^5^ and 10^6^ CFU/g.

## 4. Conclusions and Future Implications

The long shelf life of our food items, inadequate temperature control, abuse at the household level, combined with the ability of *L. monocytogenes* to grow at refrigeration temperatures and its ability to enter the milk chain at almost every stage, makes *L. monocytogenes* a significant threat to public health. In the context of direct marketing of raw milk and cheese, we can conclude that prudent steps must be taken by the farmers to eliminate major contamination routes, to ensure continuous compliance with the legally prescribed cooling temperature and to offer products with a short shelf life.

European Union legislation requires that food business operators not only comply to basic rules of hygiene (Good Hygiene Practices) [24,27] but, more specifically, in Article 5 of Regulation (EC) No 852/2004, “shall put in place, implement and maintain a permanent procedure or procedures based on the HACCP principles”. More recently, the establishment of “Operational Prerequisite Control Programs” (oPRP) has filled a gap between Good Hygiene Practices (GHP) and HACCP-based procedures [139]. In this context, examination of the udder and determination of the somatic cell count (CMT) of the milk are measures to detect animals with clinical or subclinical mastitis and to discard milk from such infected animals. In fresh cheese making, the addition of appropriate starter cultures can prevent multiplication of *L. monocytogenes*, or even reduce their numbers. Adequate sanitation of the milk processing area is one of the basics in GHP. However, under real-life conditions *L. monocytogenes* is sometimes able to persist in dairy plants, with severe consequences [140].

From the references we retrieved, it is obvious that in some cases, non-compliance to GHP and a lack of HACCP-based procedures were identified as factors creating hazardous situations. We cannot conclude that strict adherence to food safety management programs would render a 100% safe food. Thus, risk management by a shift towards heat-processed products would more likely allow a fully HACCP compliant food safety system for control of *L. monocytogenes* on small on-farm dairies.

## Data Availability

No new data were created or analyzed in this study. Data sharing is not applicable to this article.
